# Bubble-mediated hydrogen degassing in the rumen: a theoretical hypothesis with potential implications for methane mitigation

**DOI:** 10.3389/fmicb.2026.1903007

**Published:** 2026-08-18

**Authors:** Stephen J. Sowerby

**Affiliations:** Rumenaut Limited, Dunedin, New Zealand

**Keywords:** aerophilic, classical nucleation theory, dissolved hydrogen, methanogenesis, rumen

## Abstract

The rumen is a fluidic system in which dissolved gases routinely exceed their Henry's Law equilibrium concentrations and are ultimately eructed, implying the existence of physical degassing processes. However, the mechanisms governing intraruminal degassing are poorly characterized. In this study, a theoretical hypothesis is proposed based on thermodynamic and mechanistic considerations, in which bubble-mediated degassing competes with methanogenesis for dissolved hydrogen (H_2_). This hypothesis further contemplates intraruminal devices incorporating engineered surfaces that could promote bubble formation and potentially augment natural degassing pathways and inhibitor-based methane (CH_4_) mitigants that elevate dissolved hydrogen. This approach integrates physical chemistry, interfacial science, and rumen microbiology and is amenable to evaluation using established methods. Accelerating the transition of excess dissolved hydrogen into the gas phase may provide a new physical mechanism for enteric methane mitigation.

## 1 Introduction

Methane (CH_4_) emitted by ruminant animals represents a loss of metabolic energy and is a potent greenhouse gas that contributes substantially to anthropogenic climate change (Johnson and Johnson, 1995; Moss et al., 2000; Thorpe, 2009). Methanogenic archaea consume rumen hydrogen (H_2_) to facilitate the enzyme-catalyzed stepwise reduction of carbon dioxide (CO_2_), which is the predominant source of ruminant CH_4_ production (Khairunisa et al., 2023). In addition to methanogenesis, rumen H_2_ supports diverse hydrogen-consuming pathways, including acetogenesis, nitrate reduction, sulfate reduction, and fumarate metabolism (Greening et al., 2019; Khairunisa et al., 2023). Existing CH_4_ mitigation strategies are diverse (Ungerfeld and Pitta, 2024), and the inhibition of methanogenesis results in increased H_2_ emissions (Hristov et al., 2015; Lopes et al., 2016; Roque et al., 2021; Fennessy et al., 2025). In the rumen, liquid-dissolved H_2_ (dH_2_) and other dissolved gases often exceed their Henry's Law equilibrium concentrations (Janssen, 2010; Laporte-Uribe, 2016; Wang et al., 2016). Supersaturation and the eructation of rumen gases together imply that degassing of the liquid phase must occur. However, despite extensive scientific knowledge of liquid degassing, the literature appears silent on this process in the ruminant gut and has received no observable attention in methane mitigation research. Here, gas bubble formation is proposed as a natural mechanism of rumen degassing, and a brief overview of contemporary bubble theory is outlined. A novel theoretical hypothesis is proposed: bubble-mediated degassing of dH_2_ competes directly with methanogenesis and other dH_2_-dependent pathways and can be augmented through the administration of intraruminal devices comprising surfaces that promote heterogeneous bubble nucleation.

## 2 Intraruminal degassing hypothesis

### 2.1 The rumen, rumen gases, and Henry's Law

**Figure 1:**
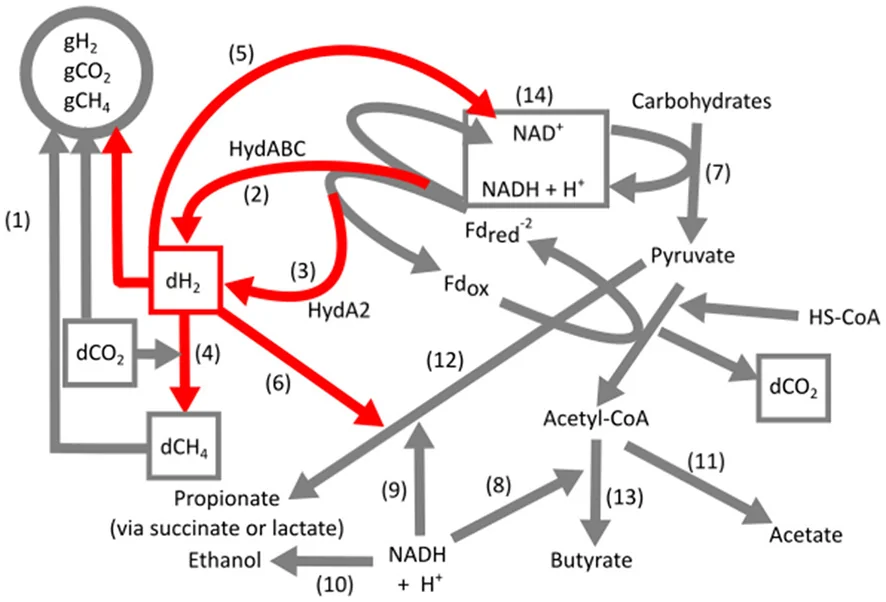
Depiction of fermentation and gas dynamics in the rumen, adapted from Ungerfeld and Pitta (2024) under a Creative Commons CC BY License. Key labeled features include dissolved-to-gaseous phase transitions (1), dH_2_ flows in red (1–5), dH_2_-dependent thermodynamic control (5, 6), NADH-coupled reactions (2, 3, 5, 7–10), volatile fatty acid anabolism (11–13), and NADH recycling to NAD^+^ (14 by 5). Pathways associated with dH_2_ excess that are upregulated include methanogenesis (4) and propionate production (12 by 6), whereas NADH recycling to NAD^+^ (14) is downregulated (5), causing down-regulation of acetate (11) and butyrate (13) production. Inhibition of methanogenesis blocks a key dH_2_ sink at (4) and causes intraruminal dH_2_ to rise and exacerbate the effects of excess dH_2_. Reducing dH_2_ by degassing (1) is predicted to have the opposite effects: Reduction of methanogenesis (4), upregulation of NADH to NAD^+^ recycling (14), increases in acetate and butyrate, and reduced propionate production (12 by 6). Symbols and abbreviations: CoA, coenzyme A; dCO_2_, dissolved carbon dioxide; gCO_2_, gaseous carbon dioxide; dH_2_, dissolved hydrogen; gH_2_, gaseous hydrogen; dCH_4_, dissolved methane; gCH_4_, gaseous methane; Fd_ox_, ferrodoxin oxidized; ${\text{Fd}}_{\text{red}}^{-2}$, ferrodoxin reduced; HydA2, ferrodoxin-dependent prototypical hydrogenase; HydABC, ferrodoxin-dependent and NAD-dependent bifurcating hydrogenase; NAD^+^, oxdised nicotinamide adenine dinucleotide; NADH, reduced nicotinamide adenine dinucleotide.

The rumen is the first and largest repository of ingested feed in the ruminant foregut, containing a liquid-bathed digestate with a complex structure and rheology (Lampila and Poutiainen, 1966; Cheng and McAllister, 1997; Lentle and Janssen, 2008; Hummel et al., 2009), and is populated by a consortium of diverse microorganisms (Mizrahi et al., 2021; Perez et al., 2024). The digestate facilitates the microbiological breakdown of cellulosic plant polymers into simple sugars and the fermentative production of a homologous family of short-chain fatty acids (SCFAs), also known as volatile fatty acids (VFAs) (Bergman, 1990). These energy-yielding molecules are absorbed across the rumen wall into the bloodstream, where different VFAs are used selectively by the animal in a tissue-specific way for growth and metabolism and have also been implicated in the regulation of feed intake (Elliot et al., 1985; Bergman, 1990; Farningham and Whyte, 1993; Oba and Allen, 2003). By-products of rumen fermentation include the molecular gases carbon dioxide (dCO_2_) and hydrogen (dH_2_), which are initially produced dissolved in the liquid phase and are identified here with the prefix “d.” Figure 1 shows intraruminal fermentation and gas dynamics: dH_2_ is predominantly produced through the action of ferrodoxin-dependent hydrogenase enzymes and is coupled to the recycling of the reduced cofactor nicotinamide adenine dinucleotide (NADH) to its oxidized form, NAD^+^; dH_2_ is predominantly consumed through the oxidative action of diverse hydrogenase enzymes in hydrogenotrophic organisms; the concentration of dH_2_ thermodynamically controls cofactor recycling, causing feedback inhibition when elevated; dH_2_ is normally maintained at low levels by hydrogenotrophic methanogens that combine dH_2_ with dCO_2_ to synthesize methane (dCH_4_); and when dH_2_ levels are low, hydrogenase expression in other hydrogenotrophic organisms is upregulated. However, when methanogenesis is inhibited, dH_2_ concentrations increase significantly, reshaping the VFA profile, fermentation efficiency, energy dynamics, and microbial composition (Greening et al., 2019; Ungerfeld, 2020; Ungerfeld and Pitta, 2024). Therefore, promoting the removal of dH_2_ from the rumen and thereby reducing dH_2_ concentrations presents the potential to reduce methanogenesis and upregulate other hydrogen-consuming pathways.

It is generally accepted that dissolved gases are the source of their gaseous-phase counterparts, gCO_2_, gH_2_, and gCH_4_, identified here with the prefix “g” (Theodorou et al., 1998; Janssen, 2010; Laporte-Uribe, 2016; Wang et al., 2016). These digestate gases in the rumens of grazing ruminants ultimately accumulate in the dorsal sac, forming a gas dome (Weiss, 1953; Hoffmann, 1973; Clauss et al., 2003). Increasing intraruminal gas pressure triggers the animal's eructation reflex. This muscular contraction causes the accumulated gases to be pushed from the gas dome into the esophagus (Weiss, 1953) and subsequently emitted into the atmosphere via the mouth and nostrils (Jaimes et al., 2025). The gaseous compounds gCO_2_, gH_2_, and gCH_4_, together with nitrogen (gN_2_), oxygen (gO_2_), hydrogen sulfide (gH_2_S), and nitrous oxide (gN_2_O), can all be measured in the emission profile (Min et al., 2022). Nitrogen and oxygen have been attributed to swallowed air (McAnally and Phillipson, 1944).

The rumen is a thermodynamically open system with episodic exchanges of solid, liquid, and gaseous matter. The normal temperature is approximately 39 °C (312 K), but it exhibits sharp temperature fluctuations due to the ingestion of water and feed (Welsh et al., 2025). Pressure measured using intraruminal devices has a baseline of ~105 kPa, with periodic increases to ~110 kPa during ruminal contractions and eructation (Sievers et al., 2004; Bishop-Hurley et al., 2016). The adult bovine rumen contains a liquid volume of 50–100 L (Moloney et al., 1993) and generates a total eructation gas volume of 30–60 L/h (Colvin et al., 1957). This corresponds to 500–1,000 mL/min or 8–17 mL/s, but it is presently unknown what proportion of this volume can be attributed to any specific gas-generating mechanism.

The key rumen gases by composition and metabolic role are dCO_2_, dH_2_, dCH_4_, dN_2_, and dO_2_. They all have zero dipole moments (Lide, 2005) and are therefore non-polar and exhibit low aqueous solubility (Table 1). Dissolved gas molecules exclude water from the volumes they occupy, and water molecules adopt configurations that surround the solutes without breaking hydrogen bonds (Chandler, 2005).

**Table 1:** Selected properties of rumen gases.

Gas	Rumen emission range (~%)^a^	$H_{i}^{cp}$ (c_aq_/p_g_) (mol/m^3^/Pa)^b^	$H_{i}^{cc}$ (c_gas_/c_aq_)^b^	Solubility χ_*i*_^c^	Solubility *C*_*i*_ (mM)^d^	Diffusivity *D*_*i*_ (x10^−9^ m^2^/s)^e^	Density ρ_*g*_ (kg/m^3^)^f^
CO_2_	45–75	3.3 × 10^−4^	1.19	6.15 × 10^−4^	34.0	1.91	1.84
CH_4_	20–30	1.4 × 10^−5^	28.81	2.55 × 10^−5^	1.41	1.84	0.67
N_2_	7.0	6.4 × 10^−6^	63.03	1.18 × 10^−5^	0.66	2.00	1.17
O_2_	0.5	1.2 × 10^−5^	31.03	2.29 × 10^−5^	1.27	2.42	1.33
H_2_	0.2	7.8 × 10^−6^	51.72	1.41 × 10^−5^	0.78	5.11	0.09
H_2_S	Trace	1.0 × 10^−3^	0.40	1.85 × 10^−3^	102.4	1.36	1.43
N_2_O	Trace	2.4 × 10^−4^	1.68	4.37 × 10^−4^	24.3	2.57	1.83

^a^Min et al., 2022.

^b^Sander, 2023.

^c^At 298.15 K and with all gases at a partial pressure of 101.325 kPa (Gevantman, 1995).

^d^Calculated from c.

^e^At 298.15 K and 101.325 kPa (The Engineering Toolbox, 2008).

^f^At 273.15 K and 101.325 kPa (The Engineering Toolbox, 2003).

Rumen gases have often been quantified using Henry's Law (Theodorou et al., 1998; Janssen, 2010; Laporte-Uribe, 2016; Wang et al., 2016), which describes the thermodynamic equilibrium between liquid-dissolved and gaseous-phase species and is expressed as follows:


$$\begin{array}{l} C_{i}^{\text{eq}}=H_{i}^{cp}P_{i}^{\text{gas}} \end{array}$$

where $C_{i}^{\text{eq}}$ is the equilibrium concentration of dissolved gas species *i*, $P_{i}^{\text{gas}}$ is the partial pressure of the ga*s*, and $H_{i}^{cp}$ is the Henry constant (Sander, 2023). The Henry constant can be quantitatively expressed as a solubility constant ${H_{i}}^{cp}$, where $C_{i}^{\text{eq}}$ (mol/m^3^) is divided by $P_{i}^{\text{gas}}$ (Pa) and has units of mol/m^3^/Pa. The unitless Henry volatility constant ${H_{i}}^{cc}$ represents the molar ratio between the gaseous and the dissolved-phase species (see Table 1) and provides a volatility ranking for the dissolved gases:


$$\begin{array}{l} dN_{2}≳dH_{2}>dO_{2}≳dCH_{4}\gg dCO_{2} \end{array}$$

Supersaturation is a state where the empirically determined dissolved gas concentration is greater than that predicted by Henry's Law from the gaseous phase partial pressure. A saturation ratio, *S*_*i*_, for species *i* can be defined by the following expression:


$$\begin{array}{l} S_{i}=\frac{C_{i}^{\text{meas}}}{C_{i}^{\text{eq}}} \end{array}$$

where $C_{i}^{meas}$ is the measured concentration of the dissolved gas. From paired measurements of gH_2_/dH_2_ and gCH_4_/dCH_4_ obtained directly from live animal rumen sources, both gas pairs were found to be significantly higher than Henry's Law predictions and yielded supersaturation ratios of 38 to 43 for dH_2_ and 6 to 8 for dCH_4_ (Wang et al., 2016). Rumen liquid also regularly exceeds the saturation levels of dCO_2_ (Laporte-Uribe, 2016). Although an equilibrium approach to gas partitioning describes the thermodynamic drivers of phase transitions, it provides no insight into the underlying mechanisms or kinetics.

**Figure 2:**
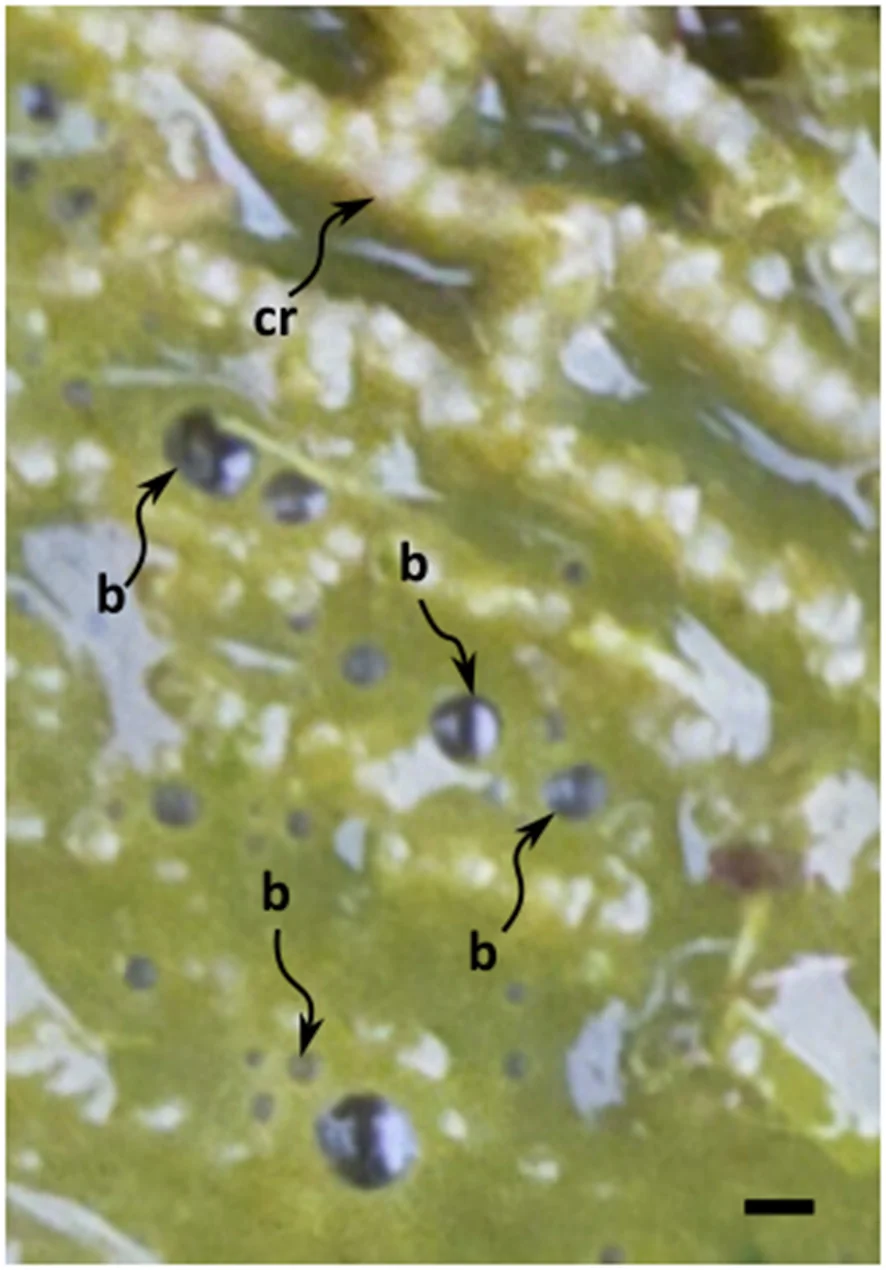
Photograph of a portion of a freshly dissected reticulorumen from a fallow deer (*Dama dama*). Arrows indicate cristae reticuli (cr) and gas bubbles (b). The scale bar in the lower left represents 1 mm.

The pathology of bloat as a barrier to rumen degassing has received considerable attention (Kleiber et al., 1943; Clarke and Reid, 1974; Moate et al., 1997; Wang et al., 2012). However, surprisingly little attention has been given to the mechanism governing degassing in the normal, healthy rumen. Gas bubbles have been observed in the rumen digestate (Hungate, 1975; Prins and Riet, 1987), and mixing in anaerobic digesters occurs via bubble adherence to microparticles (Mason and Stuckey, 2016). Gas bubbles are reported to form and rise through the digestate and coalesce with the gas cap in the dorsal sac (Clarke and Reid, 1974). Bubble-mediated degassing in many natural and industrial settings has a rich theoretical and experimental history (Epstein and Plesset, 1950; Blander and Katz, 1975; Prausnitz et al., 1999; Chandler, 2005; Vehkamäki, 2006; Sear, 2007; Liger-Belair, 2012; Gallo et al., 2020). Figure 2 shows gas bubbles in the liquid phase of a freshly dissected reticulorumen, providing direct evidence that gas bubbles exist within the rumen liquid phase. Accordingly, it is reasonable to explore the mechanisms by which bubble formation may facilitate degassing of the live healthy rumen.

### 2.2 Bubble theory

#### 2.2.1 Bubble formation

The nature of bubble formation can be addressed by classical nucleation theory (CNT), which applies to the formation of bubbles, droplets, and crystals. CNT provides a thermodynamic framework for understanding when and how embryos of a new phase first appear within a parent phase (Blander and Katz, 1975; Vehkamäki, 2006; Sear, 2007; Gallo et al., 2020). As with all mathematical abstractions of real-world phenomena, equations necessarily represent simplifications. Bubble formation, defined by CNT as the creation of a new interface enclosing a volume of the new gas phase, does not account for the full complexity of rumen fluids; however, it provides a framework for examining the factors potentially relevant to interfacial processes within the rumen.

Creating a new bubble requires free energy, whereas growing an existing bubble releases free energy. In thermodynamic terms, the nucleation barrier associated with forming a new bubble embryo of radius *r* within a liquid is represented by the Gibbs free energy change (Δ*G*_*r*_), which is expressed as:


$$\begin{array}{l} \Delta G_{r}=4\pi r^{2}\gamma_{LG}-\frac{4}{3}\pi r^{3}\Delta p \end{array}$$

where *γ*_*LG*_ is the surface tension of the liquid–gas interface. The term Δ*p* = *p*_*gas*_ − *p*_*liquid*_ reflects the thermodynamic driving pressure between the bulk liquid and the gas phase, which is alternatively called the supersaturation pressure difference. The nucleation barrier represents the high cost of breaking hydrogen bonds to create a sufficiently large spherical cavity, where the interfacial curvature prevents water molecules from maintaining their full hydrogen-bonding with the surrounding network (Chandler, 2005).

CNT predicts the critical bubble radius r^*^, which is governed by the balance between the surface tension of the liquid–gas interface *γ*_*LG*_ and the pressure difference across that interface and is expressed as follows:


$$\begin{array}{l} r^{*}=\frac{2\gamma_{LG}}{\Delta p} \end{array}$$

Only bubbles larger than r^*^ will persist, whereas smaller bubbles will be unstable and collapse.

In *homogeneous* nucleation, a bubble embryo forms entirely within the bulk liquid. A full spherical interface area of 4*πr*^2^ must be created instantaneously, making Δ*G*_*r*_ extremely large due to the high surface tension of water. The height of the nucleation barrier for homogeneous nucleation Δ$G_{\mathrm{hom}}^{*}$ is given by:


$$\begin{array}{l} \Delta G_{\mathrm{hom}}^{*}=\frac{16\pi \gamma^{3}}{{3\left(\Delta p\right)}^{2}} \end{array}$$

In the process of *heterogeneous* nucleation, a solid surface lowers the nucleation barrier by providing an interface for solvated gas molecules to contact, desolvate, and adsorb. On a submerged surface, an embryonic gas bubble is a spherical cap rather than a full sphere and the barrier for heterogeneous nucleation $\Delta G_{het}^{*}$ is reduced according to the following equation:


$$\begin{array}{l} \Delta G_{\text{het}}*=f\left(\theta \right)\Delta G_{\text{hom}}^{*} \end{array}$$

where *f* (*θ*) is a purely geometric factor representing the volume fraction of the cap *V*_*cap*_ compared to that of a sphere *V*_*sphere*_ at the critical radius (Cabriolu and Li, 2015) and is given by:


$$\begin{array}{l} f\left(\theta \right)=\frac{V_{cap}}{V_{sphere}}=\frac{1}{4}\left(2+cos\theta \right){\left(1-cos\theta \right)}^{2} \end{array}$$

Here, *θ* is the gas-side contact angle *θ*_*G*_ of a captive bubble. The contact angle *θ*_*G*_ is empirically determined and arises from the equilibrium balance of surface forces (tensions *γ*) at the intersection between solid, liquid, and gas phases. It is derived using Young's equation (Young, 1805; George et al., 2017):


$$\begin{array}{l} \cos\theta_{G}=\frac{\gamma_{SL}-\gamma_{SG}}{\gamma_{LG}} \end{array}$$

where *γ*_*SG*_ is the surface tension of the solid–gas interface, *γ*_*SL*_ is the surface tension of the solid–liquid interface, and *γ*_*LG*_ is the surface tension of the liquid–gas interface. It is important to note that a low surface tension implies thermodynamically favorable phase contact because it reduces energy.

**Figure 3:**
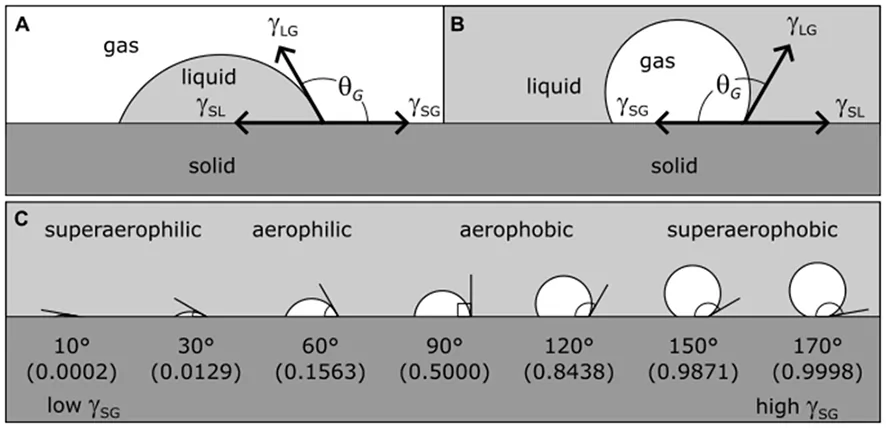
Profile drawings depicting: **(A)** a sessile drop showing the gas-side contact angle *θ*_*G*_; **(B)** a captive bubble showing the gas-side contact angle *θ*_*G*_; and **(C)** captive bubbles due to increasing surface energy *γ*_*SG*_ (left to right) generating increasing gas-side contact angles *θ*_*G*_ (10° to 170°), with their corresponding geometric factors [*f*(*θ*)] shown in parentheses.

From the static geometry of sessile droplets on dry surfaces (Figure 3A) and captive bubbles on submerged surfaces (Figures 3B, C), the wetting behavior of solids by liquids and gases can be classified and shown to be generally equivalent (George et al., 2017). When the gas-side contact angle is $\theta_{G}\geq 90^{•}$, a high-energy surface is both hydrophilic and aerophobic and is preferentially wetted by water. In contrast, when $\theta_{G}<90^{•}$, a low-energy surface is both hydrophobic and aerophilic and is preferentially wetted by gas (Figure 3C). It follows that high-energy surfaces with large *γ*_*SG*_ and small *γ*_*SL*_ generate a large *θ*_*G*_ and a large *f* (*θ*), whereas low-energy surfaces with small *γ*_*SG*_ and large *γ*_*SL*_ generate a small *θ*_*G*_ and a small *f* (*θ*). Gas adsorbed on a low-energy surface under liquid minimizes the total interfacial free energy by increasing its gas–solid contact area and reducing its liquid–gas area. The geometric factor *f* (*θ*) is the multiplier by which the heterogeneous nucleation barrier is reduced. Gas adherence to hydrophilic or aerophobic surfaces would require a larger liquid–gas interface, which is energetically expensive and therefore does not persist in practice. Figure 3C depicts exemplary bubble geometries and their corresponding *f* (*θ*) values.

Microscopic surface features that introduce concave geometries, including defects, crevices, and pits that trap gas, can produce a small *θ*_*G*_ and lead to a small *f* (*θ*). Such localized features can act as strong nucleation sites because they reduce the bubble nucleation barrier by minimizing the solid–liquid contact area (Jones et al., 1999). Surface textures comprising multiple such air-trapping features, or more generally mixtures of low- and high-energy surface patches, can generate very small contact angles (*θ* ≤ 10°) and are classified as both superhydrophobic and superaerophilic.

Microscopically textured surfaces can be homogeneously wetted (the Wenzel state) or can trap gas within surface microcavities, leading to heterogeneous wetting (the Cassie-Baxter state), and both states can be treated as extensions of Young's equation (McHale, 2007). Some such surfaces, when submerged, can retain a thin plastron of gas to which nothing else adheres (Dhyani et al., 2021; Tesler et al., 2024). It is sufficient to note here that low-energy surfaces and high-energy surface features can both lower the bubble nucleation barrier.

A consequence of the nucleation barrier being a free-energy barrier is that it allows prediction of the nucleation rate, *J*, which follows an Arrhenius-type expression:


$$\begin{array}{l} J=J_{0}\text{exp}\left(-\frac{f\left(\theta \right)\Delta G_{\mathrm{hom}}^{*}}{k_{B}T}\right) \end{array}$$

where *J*_0_ is a pre-exponential kinetic factor reflecting the nucleation-attempt frequency, *k*_*B*_ is the Boltzmann constant, and *T* is the absolute temperature (Kashchiev and Rosmalen, 2003). What emerges is that the rate of heterogeneous gas bubble nucleation is strongly dependent on the contact angle: The lower the gas-side contact angle, the smaller the geometric factor, the lower the nucleation barrier, and the higher the nucleation rate.

#### 2.2.2 Bubble growth and composition

The gas-side region of the water–gas interface constitutes an extremely hydrophobic environment that favors the adsorption of non-polar solutes (Hummer et al., 1998; Chandler, 2005; Oss et al., 2005). Dissolved gas molecules diffuse toward the interface, where their solvated structure becomes asymmetric, and the surrounding hydrogen-bonded network is destabilized (Hummer et al., 1998). As the free energy of a hydrophobic solute is lower at the interface than in bulk liquid, the dissolved gas molecule can desolvate and cross into the low-density gas phase, where relocation is thermodynamically favorable. Once a gas nucleus is present, its subsequent growth or dissolution is governed by diffusion.

The Epstein–Plesset equation describes the rate of change of a growing bubble radius *R*(*t*) and is given by:


$$\begin{array}{l} \frac{dR}{dt}=\frac{D\left(C_{\infty }-C_{s}\right)}{\rho_{g}R}\left(1+\frac{R}{\sqrt{\pi Dt}}\right) \end{array}$$

where *D* is the diffusion coefficient (diffusivity) of the dissolved gas, *C*_∞_ is the gas concentration far from the bubble, *C*_*s*_ is the concentration at the bubble surface, and *ρ*_*g*_ is the gas density inside the bubble (Epstein and Plesset, 1950; Su and Needham, 2013). The first term reflects the thermodynamic drive: if *C*_∞_ > *C*_*s*_, the liquid is supersaturated and the bubble grows; if *C*_∞_ < *C*_*s*_, the liquid is undersaturated and the bubble dissolves. The diffusivity relates to the dissolved gas mobility through the surrounding liquid, and *ρ*_*g*_*R* reflects the mass-to-radius conversion. The last term represents the time-dependent unsteady-diffusion correction as the bubble radius changes and captures the initial rapid growth of bubbles.

A key feature of bubble growth is that the radius grows proportionally to the square root of time when diffusion is the rate-limiting process. The interface absorbs gas rapidly, so the rate-determining process is diffusion through the surrounding liquid (Enríquez et al., 2013; Maloth et al., 2022). A bubble can only grow as rapidly as dissolved gas can diffuse through the liquid to its surface. As shown in Table 1, the relative diffusivities of the dissolved rumen gases exhibit the following diffusivity (mobility) ranking:


$$\begin{array}{l} D_{dH_{2}}\gg D_{dO_{2}}>D_{dN_{2}}>D_{dCO_{2}}>D_{dCH_{4}} \end{array}$$

As a bubble grows, it extracts dissolved gas from the surrounding liquid, creating a concentration gradient and a depletion zone that causes the diffusive flux into the bubble to decrease slowly (Soto et al., 2017). Consequently, in a liquid containing multiple species of dissolved gas, different gases with different diffusivities create distinct depletion zones, which evolve at different rates.

The bubble composition also evolves according to the thermodynamic drive of the various species that transition into the gas phase (Rezvani and Elliott, 2025). The partial molar Gibbs free energy of gas species *i*, also called the chemical potential *μ*_*i*_, determines whether a dissolved gas species leaves the liquid phase and enters the gas phase. From thermodynamic definitions (Prausnitz et al., 1999; Atkins et al., 2022), the general expression for the chemical potential *μ*_*i*_ of species *i*, applicable to both gaseous $\mu_{i}^{g}$ and dissolved $\mu_{i}^{l}$ states, is:


$$\begin{array}{l} \mu_{i}=\mu_{i}^{•}+RTlna_{i} \end{array}$$

where $\mu_{i}^{•}$ is the standard chemical potential, *a*_*i*_ is the chemical activity (the *effective* concentration), *R* is the universal gas constant, and *T* is the absolute temperature. For ideal gases, $a_{i}=p_{i}/p^{•}$, where *p*_*i*_ is the partial pressure of species *i* and *p*° is the standard pressure (1 bar). For ideal solutions, *a*_*i*_ = *c*_*i*_/*c* = χ_*i*_, where *c*_*i*_ is the concentration of species *i*, *c* is the standard concentration (1 mol/L), and χ_*i*_ is the mole fraction. For real gases, *a*_*i*_ = *f*_*i*_/*p*, where *f*_*i*_ is the fugacity of species *i*. For real solutions, *a*_*i*_ = *γ*_*i*_*c*_*i*_/*c* = *γ*_*i*_*χ*_*i*_, where *γ*_*i*_ is an activity coefficient of species *i*. Under supersaturation conditions, *a*_*i*_ reduces to $S_{i}=C_{i}^{meas}/C_{i}^{eq}$.

If $\mu_{i}^{l}>\mu_{i}^{g}$, dissolved gas species *i* flows into the gas phase (bubble). Accordingly, if $\mu_{i}^{l}<\mu_{i}^{g}$, species *i* flows from the gas phase into the liquid phase. If $\mu_{i}^{l}\approx \mu_{i}^{g}$, the phases are in equilibrium and there is no net transfer of gas species. These relationships link $\mu_{i}^{g}$ to its partial pressure inside the bubble and $\mu_{i}^{l}$ to its dissolved concentration in the surrounding liquid.

The combination of the Henry volatility constants and the supersaturation ratios reported for dH_2_ and dCH_4_ (Wang et al., 2016) predict a provisional liquid-phase chemical potential ranking, contingent on the paired gaseous and dissolved concentrations of all rumen gases, as follows:


$$\begin{array}{l} \mu_{H_{2}}^{l}>{\mu_{CH_{4}}^{l}>\mu }_{N_{2}}^{l}>\mu_{O_{2}}^{l}>\mu_{CO_{2}}^{l} \end{array}$$

When dissolved gas molecules cross the liquid–gas interface, they push into the volume of the bubble interior and increase the number of molecules in the gas phase. The surface tension remains constant, so the bubble must expand to accommodate the additional gas content. There are strong radius- and curvature-dependent effects. The Laplace pressure Δ*P* is a mechanical consequence of the curvature of the interface that arises from the minimization of interfacial surface energy (Siqveland and Skjaeveland, 2021). Very small bubbles have very high curvature and a large Laplace pressure, which increases the gas-phase chemical potential $\mu_{i}^{g}$ inside the bubble. Only dissolved gases with sufficiently high liquid-phase chemical potentials, $\mu_{i}^{l}$, will contribute to bubble growth. Consequently, the nascent bubble composition will be dominated by gases positioned toward the left side of the liquid-phase chemical potential ranking. As the bubble radius increases, the Laplace pressure decreases, the interface area increases, the curvature is reduced, and other dissolved gases will add to the bubble composition. Consequently, because dH_2_ has one of the highest chemical potentials ($\mu_{i}^{l}$) and the highest diffusivity (*D*) (2 to 3 times faster than other dissolved rumen gases), the early bubble composition is predicted to be enriched in gH_2_ relative to the dissolved-phase species concentrations.

#### 2.2.3 Bubble detachment and passage

A captive bubble remains attached to a surface until its upward net buoyancy force exceeds the vertical component of its adhesion force. There are many models for bubble attachment, detachment, and rise in fluids (Jones et al., 1999; Thorncroft and Klausner, 2002; Bothe et al., 2025; Dao et al., 2025). However, rumen contraction-induced mixing of the digestate will inevitably cause collisions between particles and the generation of shear forces at solid–liquid interfaces. This mechanical action may augment buoyancy-driven bubble release and result in smaller and more frequent bubble detachment events. The complexity of the rumen digestate challenges conventional bubble adhesion and passage models: The rumen is highly compartmentalized (Lampila and Poutiainen, 1966; Cheng and McAllister, 1997); the stratification of liquids, solids, and gases within the digestate differs according to animal feeding type (Clauss et al., 2003; Tschuor and Clauss, 2008); and the rheological properties change according to feed composition and time after feeding (Hummel et al., 2009; Lentle et al., 2010). Nevertheless, the formation of a gas dome in the dorsal sac of the rumen and the eructation of rumen gases are characteristic features of grazing ruminants and indicate the presence of buoyancy-driven gas stratification processes.

## 3 Implications for natural and augmented rumen degassing

### 3.1 Natural rumen degassing

Little is known about the natural degassing processes in the rumen. In frothy bloat, the formation of stable foams arising from feed components prevents rumen gas escape. This can be remediated through the administration of surface tension-reducing agents (Clarke and Reid, 1974) and demonstrates the feed-dependent sensitivity to interfacial properties and rumen degassing. Surface tension and the physical parameters of the gases pervade the mathematical functions presented here. However, surface tension measurements are notoriously difficult to perform, lack reliability, and are highly dependent on the precise material composition and measurement technique (Oosterlaken and With, 2022). Furthermore, all standard liquid-phase properties of the gases listed in Table 1 are likely to be modulated by feed composition, the macroscopic and microparticulate components of fiber digestion, the molecular products of microbial activity, and the presence of surfactants and inorganic species. Consequently, actual intraruminal performance metrics and biological significance will be difficult to predict from first principles and must be calibrated through empirical measurements.

Rumen digestate materials are predominantly cellulosic and generally hydrophilic, with high surface energy (Luner and Sandell, 1969), but they also contain hydrophobic domains (Cosgrove, 2014). Up to 70% of leaf structure comprises gas-filled cavities (Earles et al., 2018; Whitewoods, 2021), and these structures are likely to introduce intact liquid–gas interfaces into the rumen. Such pre-existing gas pockets can provide a zero-barrier pathway for highly active degassing, analogous to the characteristic bubble streams seen in champagne. Fermented liquids are illustrative: Gas-filled microscopic capillaries of plant origin undergo interface expansion and pinching cycles that repeatedly release bubbles while re-establishing the liquid–gas interface (Liger-Belair, 2012). Native formation of gas bubbles in the rumen may also occur through heterogeneous nucleation on the high-energy fracture edges generated by fiber mastication (Pérez-Barbería and Gordon, 1998) and on the hydrophobic domains of the material surface. The subsequent bubble history, comprising nucleation, growth, detachment, and ultimate passage to the gas dome, will likely be shaped by factors including pressure and temperature fluctuations; chemical composition of the liquid phase; microbial activity; interfacial factors of the rumen environment; and rumen motility, which generates collisions and shear forces.

Theory predicts that bubble compositions will reflect the rumen gas species in proportions dictated by the species-specific chemical potentials and diffusivities of the dissolved gases. Rapid bubble detachment could introduce compositional asymmetry because different gases diffuse into the bubbles at different rates. The high chemical potential and exceptionally high diffusivity of dH_2_ predict that nascent bubbles will be enriched in gH_2_. In ruminant gas emissions, gH_2_ represents a minor fraction (Table 1). However, when methanogen inhibitors are administered, gH_2_ emissions are observed to increase in a dose-related manner (Hristov et al., 2015; Lopes et al., 2016; Roque et al., 2021; Fennessy et al., 2025). On the basis of such observations, the presence of intraruminal gas bubbles, and CNT, a hypothesis is proposed: gas bubbles compete directly with methanogens, other hydrogenotrophs, and metabolic reactions for dH_2_. Furthermore, because elevated intraruminal dH_2_ has demonstrable impacts on VFA profiles, fermentation efficiency, energy dynamics, and microbial composition (Janssen, 2010; Greening et al., 2019; Ungerfeld, 2020; Ungerfeld and Pitta, 2024), bubble-mediated removal of dH_2_, is also likely to influence these dH_2_-dependent pathways.

### 3.2 Augmenting rumen degassing

Although there are many unanswered questions about native degassing in the rumen, it is compelling to consider the deliberate administration of materials to promote bubble formation and augment natural degassing processes. For exotic, low-surface-energy materials configured into a device and administered into the rumen, theory predicts the nucleation of gas bubbles with low nucleation barriers and high nucleation rates to augment native degassing mechanisms. On aerophilic surfaces, artificially generated bubbles enriched in gH_2_ molecules could remove dH_2_ from the liquid phase, alleviating inhibition of cofactor recycling, reducing detrimental effects on VFA profiles, and limiting hydrogenotrophic production of dCH_4_, while upregulating other hydrogen-consuming pathways. The reduction of increased dH_2_ caused by methanogen inhibitors could potentially abate some of the associated inhibitor side effects. The potential for artificial degassing to mitigate hydrogenotrophic methane production is compelling, but its scalability and potential off-axis effects on broader dH_2_ metabolism will require careful examination.

#### 3.2.1 Risks

The risks associated with intraruminal devices can be broadly classified into animal risks and technology risks.

##### 3.2.1.1 Animal risks

The administration of any intraruminal device carries the potential for health and productivity complications. There are potential long-term challenges associated with biocompatibility and effects on rumen motility and mucosal irritation, which may lead to unanticipated veterinary interventions such as hardware disease (Martel et al., 2021; Airs et al., 2024). Excessive degassing could detrimentally deplete dH_2_ below metabolically normal levels. If bubbles form but cannot escape, they could potentially contribute to foam formation and induce or exacerbate bloat-like pathology. For animal compatibility, examples of esophagus-administered bolus devices have demonstrated physical attributes that enable safe administration and persistent retention within the rumen (Vandamme and Rathbone, 2012; León-Cruz et al., 2020). These devices provide indicative parameters for anatomical acceptability. The potential implications for degassing the rumen of dissolved gases can only be informed by experimentation comprising carefully calibrated *in vitro* studies and ethically approved animal trials. Understanding and tuning the surface energy, texture, and area can be used to modulate degassing limits.

##### 3.2.1.2 Technology risks

Various degassing devices are conceivable, but they must address two critical issues: (1) degassing efficiency—how the material is configured to optimize degassing—and (2) animal compatibility—how the device is configured for esophageal administration and retained intact within the rumen for extended periods.

Using existing metrics and established physicochemical methods, the optimisation of degassing can be facilitated in pristine laboratory conditions using idealized, non-complex fluids. However, long-term functionality is most likely to be affected by the extreme challenges of the rumen and the formation of biofilms on active surfaces. These biological coatings (biofouling) are predicted to increase surface energy, reduce aerophilicity, and diminish the gas bubble nucleation potential. Mitigating biofouling is a critical challenge and will require suitably constructed *in vitro* experiments to test the extent and persistence of degassing functionality under relevant physiological conditions. Materials that generate superaerophilic performance are substantially more resistant to biofouling (Genzer and Efimenko, 2006) and can be predicted to facilitate accelerated degassing (Figure 3). Some engineered surfaces can repel both polar and oily materials, leading to superomniphobicity and gas plastrons (Kota et al., 2014). The prevention of biofouling has broad applications and is an active area of advanced materials research (Zhu et al., 2020; Li et al., 2025; Tesler and Goldmann, 2025). Geometric configurations informed by proven bolus technologies can guide device design for administration and placement (Vandamme and Rathbone, 2012; León-Cruz et al., 2020).

Once suitably parameterised devices have been configured, *in vivo* testing will be required to validate safety and functionality. Carefully controlled and ethically approved animal trials of the type currently employed for other methane mitigation approaches could provide a pragmatic pathway for evaluation.

## 4 Discussion

Whether generated by *de novo* nucleation or arising from pre-existing liquid–gas interfaces, it is likely that the formation of buoyant gas bubbles in the rumen digestate facilitates degassing and that these bubbles represent the natural mechanism through which dissolved gases ultimately enter the gaseous phase and are subsequently emitted via eructation. The predicted partitioning of rumen gases, particularly gH_2_, from the dissolved phase may have important implications for the health, productivity, and emissions of ruminant animals, given the central role of dH_2_ in rumen metabolic control and methanogenesis.

The concept of introducing exotic, low-surface-energy materials to augment natural degassing processes offers the potential for an alternative and complementary approach to modulating rumen microbiology and reducing methane emissions. The hypothesis is testable through established methods in physical chemistry and related disciplines, using relevant *in vitro* models and suitably powered *in vivo* trials.

## Data Availability

The original contributions presented in the study are included in the article/supplementary material, further inquiries can be directed to the corresponding author.
